# Oral aphthosis in children: baseline clinical features associated with diagnostic categories in a single-centre retrospective cohort

**DOI:** 10.1007/s00431-026-07405-4

**Published:** 2026-09-15

**Authors:** Rosario Francesco Dipasquale, Paola Sinopoli, Stefania Adessi, Antonio Pascuzzo, Romina Gallizzi

**Affiliations:** Department of Health Sciences, University of Catanzaro “Magna Graecia”, 88100 Catanzaro, Italy

**Keywords:** Oral aphthosis, Recurrent aphthous stomatitis, Children, PFAPA, Behçet disease, Paediatric rheumatology

## Abstract

We examined associations between clinical features at first paediatric rheumatology assessment and eventual benign/self-limited (Group 1) versus systemic/chronic (Group 2) diagnostic categories in children referred for recurrent oral aphthosis. We retrospectively studied 404 children evaluated from 2010 to 2024. Baseline clinical variables were documented at the first rheumatology visit before final diagnosis; follow-up assessments were performed every 3–6 months. Eight prespecified clinical features entered multivariable logistic regression. Firth and diagnosis-exclusion sensitivity analyses addressed sparse data and diagnostic-composition bias. CRP/ESR and HLA-B51 were analysed separately. The cohort included 198 males (49.0%) and 206 females (51.0%); age at symptom onset ranged from 2 months to 15 years. Group 1 included 267 patients, and Group 2 included 137. Recurrent fever (aOR, 0.25; 95% CI, 0.12–0.49) and tonsillitis/pharyngitis (aOR, 0.17; 95% CI, 0.09–0.32) were associated with lower odds of Group 2, whereas genital aphthosis was associated with higher odds (aOR, 13.08; 95% CI, 3.04–56.32). After simultaneous exclusion of PFAPA and Behçet disease spectrum diagnoses, the fever/pharyngitis associations were attenuated, and the genital aphthosis estimate became uninformative because of sparse data, while arthralgia/arthritis remained positively associated. Elevated CRP/ESR was not independently associated with Group 2; HLA-B51 positivity was more frequent in Group 2 among selectively tested patients.

*Conclusion*: In children referred for recurrent oral aphthosis, recurrent fever and pharyngitis were mainly associated with benign/self-limited diagnoses, whereas genital aphthosis was mainly associated with systemic/chronic diagnoses. These associations were largely driven by PFAPA and Behçet spectrum disease, respectively. Articular involvement showed the most consistent positive association across sensitivity analyses. These findings are descriptive, not a validated diagnostic prediction tool.

**Table:** 

**What is Known:** • *Oral aphthae are common and non-specific in childhood.* • *Associated symptoms guide diagnostic work-up and follow-up.*
**What is New:** • *PFAPA-characteristic features clustered with benign/self-limited diagnoses; genital aphthosis with systemic/chronic diagnoses.* • *After PFAPA/Behçet spectrum exclusion, these signals attenuated; articular involvement was the most consistent positive association.*

**Supplementary Information:**

The online version contains supplementary material available at https://doi.org/10.1007/s00431-026-07405-4.

## Introduction

Recurrent oral aphthosis is common in childhood and is usually benign, but it can also occur in autoinflammatory, vasculitic, gastrointestinal, connective tissue, and immunodeficiency disorders [1, 6]. Its diagnostic value therefore depends on the accompanying phenotype rather than on oral ulceration alone.

PFAPA is characterised by recurrent fever with aphthous stomatitis and often pharyngitis or cervical adenitis and generally follows a benign/self-limited course [2, 3]. By contrast, Behçet disease is a chronic multisystem disorder in which oral ulcers may precede other manifestations; paediatric-specific and international classification criteria emphasise multi-domain involvement [4, 5].

At first assessment, symptom combinations can therefore guide the intensity of investigation and follow-up, although incomplete presentations remain diagnostically challenging [1, 4, 5].

Manthiram et al. [7] identified shared genetic susceptibility among recurrent aphthous stomatitis, PFAPA and Behçet disease, supporting a Behçet spectrum framework. Aydin et al. [8] subsequently compared these phenotypes in a paediatric rheumatology cohort. We examined, in children referred for recurrent oral aphthosis, how clinical features already present at first rheumatological evaluation were associated with the diagnostic category assigned after the subsequent work-up.

## Materials and methods

### Study population and design

This single-centre, retrospective study was conducted at the Paediatric Rheumatology and Immunology outpatient clinic, U.O.C. of Specialist Paediatrics and Rare Diseases, Azienda Ospedaliera Universitaria “Renato Dulbecco”, Catanzaro, Italy.

We reviewed records of patients referred to the Catanzaro centre between September 2010 and October 2024 for recurrent oral aphthosis. The index assessment was the first Paediatric Rheumatology visit. All clinical variables in the primary analyses were present and documented at this visit, before final diagnostic classification; later manifestations were not recorded as baseline findings.

Patients underwent clinical follow-up with reassessments every 3–6 months during the observation period. At least one follow-up assessment after the index visit was required to complete the diagnostic evaluation. Exact individual follow-up duration could not be reliably reconstructed. Recurrent oral aphthosis was operationally defined as at least two episodes of oral mucosal ulceration in the preceding 12 months.

**Fig. 1:**
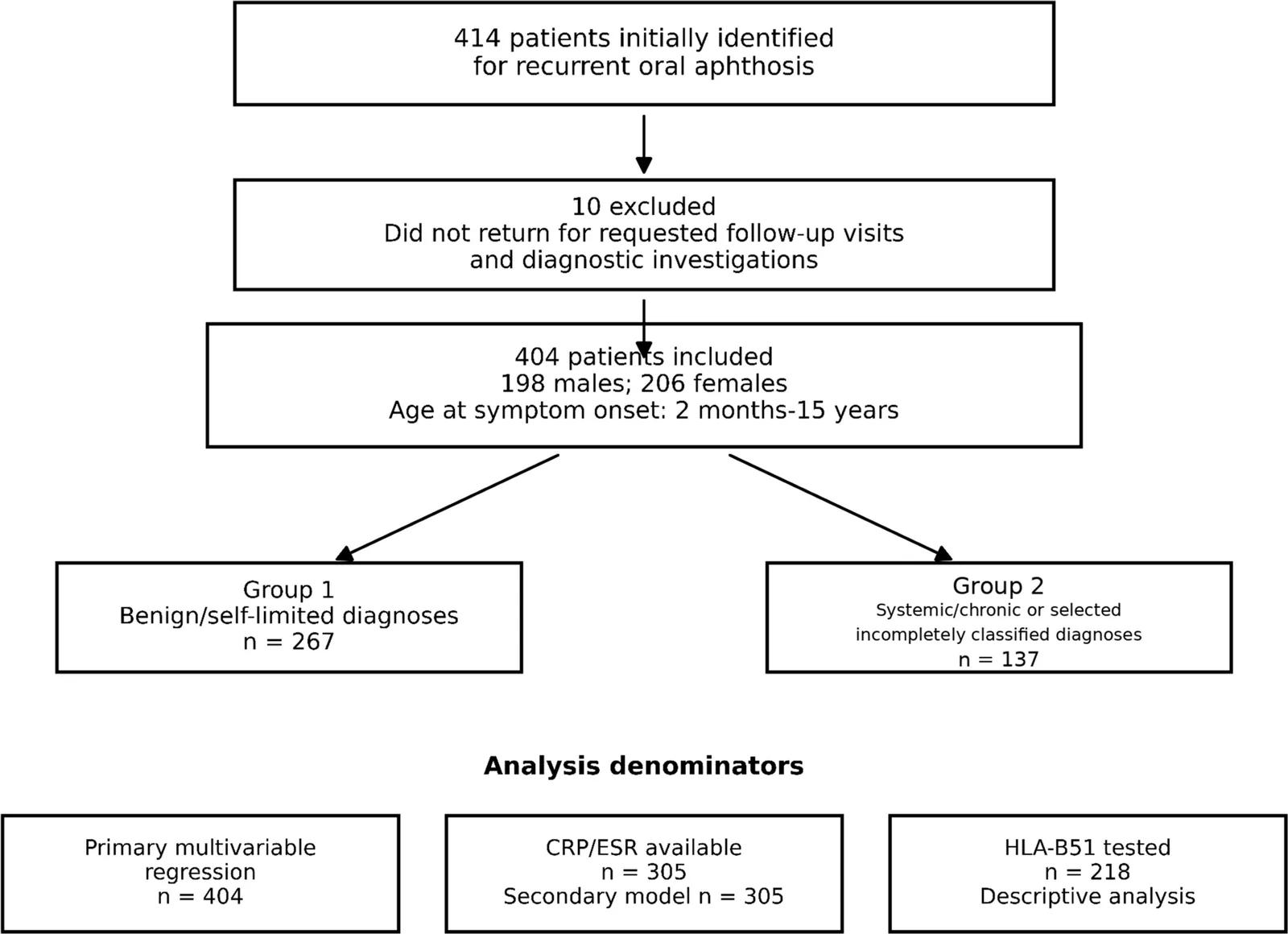
Participant flow and data availability for the main analyses

Of 414 initially identified patients, ten were excluded because they did not return to complete the requested diagnostic investigations; 404 patients were included. Participant flow and data availability for the main analyses are shown in Fig. 1.

### Data collection

Data were extracted from medical records and electronic archives. Predefined clinical variables were coded as present or absent at the index assessment. Sex and age at symptom onset were available only as aggregate descriptors for the overall cohort and were reported descriptively. Patient-level demographic variables were not retained in the analytic dataset available for this revision; consequently, group-specific age-at-onset and sex distributions and age at the index rheumatology assessment could not be reliably reconstructed. Final diagnosis and group assignment were recorded during the available follow-up.

The eight prespecified baseline clinical variables were coexistence of minor and major oral aphthae, recurrent fever, genital aphthosis, lymphadenopathy, tonsillitis/pharyngitis, arthralgia/arthritis, abdominal pain and bowel habit changes. Arthralgia and objective arthritis could not be reliably separated and were retained as a composite. Bowel habit changes comprised recurrent/persistent diarrhoea, constipation or alternating stool pattern. Oral aphthae were classified as minor (< 10 mm) or major (≥ 10 mm); the analysed variable denoted a coexistence of both phenotypes.

CRP/ESR and HLA-B51 were treated as second-level investigations. CRP/ESR was coded elevated when either exceeded the laboratory-specific upper reference limit; timing relative to febrile attacks was not standardised. HLA-B51 was requested selectively when the baseline phenotype was considered more suggestive of Behçet disease. HLA-B51 status was not used as a criterion for final diagnostic assignment, including the working diagnosis of Behçet-like disease.

Final diagnoses were stratified a priori into two pragmatic course-based categories: Group 1, benign/self-limited diagnoses, and Group 2, systemic/chronic or selected incompletely classified diagnoses. The grouping was not intended to imply shared pathophysiology within either category.

Group 1 included PFAPA (*n* = 169), recurrent fever (*n* = 33), recurrent oral aphthosis (*n* = 30), recurrent infections (*n* = 26), articular pain (*n* = 3), primary Raynaud phenomenon (*n* = 2), PFAPA-like phenotype (*n* = 2), erythromelalgia (*n* = 1) and fibromyalgia (*n* = 1). Group 2 included Behçet-like disease (*n* = 50), Behçet disease (*n* = 30), FMF (*n* = 21), juvenile idiopathic arthritis (*n* = 6), coeliac disease (*n* = 5), mevalonate kinase deficiency (*n* = 4), common variable immunodeficiency (*n* = 3), suspected FMF (*n* = 3), IgA deficiency (*n* = 2), cutaneous lupus (*n* = 2), cutaneous vasculitis (*n* = 2), and one case each of indeterminate colitis, connective tissue disease, cryopyrinopathy, secondary Raynaud phenomenon, hypogammaglobulinaemia, Crohn disease, IgA vasculitis, TINU syndrome, and TRAPS.

PFAPA was defined according to modified Marshall/Thomas clinical criteria [10]. Paediatric Behçet disease was classified primarily using PEDBD criteria, with ICBD considered supportive but not the sole determinant [4, 5]. FMF was defined using paediatric Yalçinkaya–Ozen criteria, with MEFV findings considered supportive [11]. Behçet-like disease was a specialist working diagnosis for an incomplete but clinically suggestive Behçet phenotype that did not fulfil the paediatric Behçet classification threshold during the available follow-up; the retrospective dataset did not contain sufficient domain-level detail to reconstruct an individual PEDBD/ICBD score for every such patient. PFAPA-like phenotype and suspected FMF similarly denoted clinically suggestive presentations insufficient for the complete definitions. Recurrent fever and recurrent infections denoted unclassified repeated febrile or infectious episodes after the diagnostic evaluation.

### Statistical analysis

Categorical variables were reported as *n* (%) and compared using Pearson chi-square or Fisher exact tests as appropriate. Group 2 was the outcome in multivariable binary logistic regression, including the eight prespecified baseline clinical variables simultaneously. Adjusted odds ratios (aORs) with 95% CIs were reported. Missing clinical information was not coded as absence; complete-case analysis was used. Multicollinearity was assessed using variance inflation factors (VIFs).

Because genital aphthosis had small cell counts, Firth-penalised logistic regression was used as a sensitivity analysis. Additional analyses excluded PFAPA, Behçet disease/Behçet-like disease, or both. A secondary complete-case model added CRP/ESR. HLA-B51 was analysed descriptively among the 218 tested patients, with tested-versus-untested characteristics and testing rates by diagnosis reported in Supplementary Table S1. No multiplicity correction was applied because secondary analyses assessed robustness rather than independent confirmatory hypotheses.

Conventional analyses were performed using IBM SPSS Statistics version 32.0 (IBM Corp., Armonk, NY, USA). Firth-penalised logistic regression sensitivity analyses were implemented in Python 3.13.5 using NumPy 2.3.5 and SciPy 1.17.0, using the Firth-modified score algorithm based on Jeffreys-prior penalisation. Confidence intervals were obtained from the penalised profile likelihood and *p*-values from penalised likelihood-ratio tests.

### Ethics

The study was approved by the Ethics Committee of the Azienda Ospedaliera Universitaria “Renato Dulbecco”, Catanzaro, Italy (approval number 23/2026, 21 May 2026).

## Results

Of 414 potentially eligible patients, ten did not return for the follow-up visits required to complete the requested diagnostic evaluation, leaving 404 patients (Fig. 1): 198 males (49.0%) and 206 females (51.0%), with age at symptom onset ranging from 2 months to 15 years. Group 1 comprised 267 patients (66.1%), and Group 2 comprised 137 (33.9%). PFAPA dominated Group 1 (169/267, 63.3%), whereas Behçet disease/Behçet-like disease accounted for 80/137 (58.4%) of Group 2. Baseline characteristics are shown in Table 1. All eight primary clinical variables were available for the 404 included patients.

**Table 1:** Demographic and baseline clinical characteristics according to eventual diagnostic group

Variable	Total cohort (*N* = 404)	Group 1 (*N* = 267)	Group 2 (*N* = 137)	*p*-value
Age at symptom onset, range	2 months–15 years	–	–	–
Male sex	198/404 (49.0%)	–	–	–
Female sex	206/404 (51.0%)	–	–	–
Coexistence of minor and major oral aphthae	17/404 (4.2%)	6/267 (2.2%)	11/137 (8.0%)	0.006
Recurrent fever	304/404 (75.2%)	241/267 (90.3%)	63/137 (46.0%)	< 0.001
Genital aphthosis	26/404 (6.4%)	3/267 (1.1%)	23/137 (16.8%)	< 0.001
Lymphadenopathy	119/404 (29.5%)	105/267 (39.3%)	14/137 (10.2%)	< 0.001
Tonsillitis/pharyngitis	242/404 (59.9%)	212/267 (79.4%)	30/137 (21.9%)	< 0.001
Arthralgia/arthritis	139/404 (34.4%)	77/267 (28.8%)	62/137 (45.3%)	0.001
Abdominal pain	157/404 (38.9%)	103/267 (38.6%)	54/137 (39.4%)	0.870
Bowel habit changes	62/404 (15.3%)	36/267 (13.5%)	26/137 (19.0%)	0.147

Values are *n*/*N* (%) unless otherwise indicated. Age at symptom onset and sex are reported for the overall cohort only; patient-level demographic variables were not retained in the analytic dataset available for this revision, so group-specific age-at-onset and sex distributions could not be reliably reconstructed. Pearson chi-square or Fisher exact tests were used as appropriate

In the primary model (*n* = 404), recurrent fever (aOR, 0.25; 95% CI, 0.12–0.49; *p* < 0.001) and tonsillitis/pharyngitis (aOR, 0.17; 95% CI, 0.09–0.32; *p* < 0.001) were associated with lower odds of Group 2, while genital aphthosis was associated with higher odds (aOR, 13.08; 95% CI, 3.04–56.32; *p* < 0.001). Arthralgia/arthritis showed a borderline positive association (aOR, 1.77; 95% CI, 1.000–3.126; *p* = 0.050); the remaining variables were not independently associated (Table 2). VIFs ranged from 1.04 to 1.73.

**Table 2:** Primary multivariable logistic regression of baseline clinical features

Predictor	aOR	95% CI	*p*-value
Coexistence of minor and major oral aphthae	1.58	0.46–5.42	0.471
Recurrent fever	0.25	0.12–0.49	< 0.001
Genital aphthosis	13.08	3.04–56.32	< 0.001
Lymphadenopathy	0.67	0.32–1.39	0.278
Tonsillitis/pharyngitis	0.17	0.09–0.32	< 0.001
Arthralgia/arthritis	1.77	1.000–3.126	0.050
Abdominal pain	1.58	0.85–2.93	0.149
Bowel habit changes	1.74	0.82–3.71	0.151

Group 2 is the outcome. All 404 included patients had complete data for the eight prespecified baseline clinical variables, which were entered simultaneously. For arthralgia/arthritis, the unrounded lower 95% confidence limit was 0.999733

*aOR* adjusted odds ratio, *CI* confidence interval

Firth regression produced similar estimates, with genital aphthosis remaining strongly associated (aOR, 11.06; 95% profile-likelihood CI, 3.03–51.57; *p* < 0.001) and arthralgia/arthritis remaining borderline (aOR, 1.73; 95% CI, 0.99–3.05; *p* = 0.055) (Table 3).

**Table 3:** Firth-penalised logistic regression and diagnosis-exclusion sensitivity analyses

Analysis	Recurrent fever	Tonsillitis/pharyngitis	Genital aphthosis	Arthralgia/arthritis
Full cohort (*n* = 404)	0.26 (0.13–0.50); *p* < 0.001	0.18 (0.10–0.33); *p* < 0.001	11.06 (3.03–51.57); *p* < 0.001	1.73 (0.99–3.05); *p* = 0.055
Excluding PFAPA (*n* = 235)	0.43 (0.21–0.85); *p* = 0.016	0.35 (0.17–0.70); *p* = 0.003	5.16 (1.62–21.61); *p* = 0.004	1.97 (1.07–3.71); *p* = 0.030
Excluding Behçet spectrum (*n* = 324)	0.31 (0.13–0.74); *p* = 0.008	0.25 (0.12–0.53); *p* < 0.001	0.75 (0.06–6.54); *p* = 0.797	1.91 (0.99–3.69); *p* = 0.054
Excluding PFAPA and Behçet spectrum (*n* = 155)	0.54 (0.22–1.28); *p* = 0.163	0.54 (0.23–1.24); *p* = 0.145	0.62 (0.05–4.48); *p* = 0.649	2.17 (1.06–4.46); *p* = 0.034

Values are adjusted odds ratios (95% profile-likelihood confidence intervals); *p*-value. The same eight baseline covariates are retained in each penalised model. Behçet spectrum comprises Behçet disease and Behçet-like disease

Diagnosis-exclusion analyses showed substantial case-mix dependence (Table 3). When PFAPA and Behçet spectrum diagnoses were both excluded (*n* = 155), recurrent fever and tonsillitis/pharyngitis were no longer statistically associated with Group 2, whereas arthralgia/arthritis remained positively associated (aOR, 2.17; 95% CI, 1.06–4.46; *p* = 0.034). For genital aphthosis, exclusion of Behçet spectrum diagnoses left only one exposed Group 2 patient, making the corresponding regression estimates highly imprecise and largely uninformative. Descriptively, 22 of 23 (95.7%) Group 2 patients with genital aphthosis had Behçet disease or Behçet-like disease.

In the secondary complete-case model among patients with CRP/ESR data (*n* = 305), elevated CRP/ESR was not independently associated with Group 2 (aOR, 1.49; 95% CI, 0.74–2.98; *p* = 0.264).

HLA-B51 was available in 218/404 patients (54.0%) and was requested selectively when Behçet disease was more strongly suspected. Testing proportions were similar between groups (55.1% vs 51.8%; *p*** = **0.537) but varied by diagnosis; tested and untested patients differed in recurrent fever and lymphadenopathy (Supplementary Table S1). Among tested patients, HLA-B51 positivity was more frequent in Group 2 than Group 1 (40.8% vs 4.8%; OR, 13.81; 95% CI, 5.65–33.78; *p* < 0.001). This analysis was considered descriptive and hypothesis-generating. Among three Group 1 patients with genital aphthosis, two had recurrent infections and one recurrent oral aphthosis. They lacked sufficient additional paediatric Behçet features during the available follow-up for a specialist diagnosis of Behçet disease or Behçet-like disease; the heterogeneous follow-up duration limits certainty regarding later evolution.

## Discussion

Baseline manifestations present at the first rheumatology assessment were associated with the diagnostic category during follow-up. Recurrent fever and pharyngitis were associated with Group 1, genital aphthosis with Group 2, and articular involvement showed a weaker but more persistent signal. These are adjusted associations, not a validated prediction model.

The findings fit the proposed Behçet spectrum framework. Manthiram et al. [7] demonstrated shared genetic susceptibility among recurrent aphthous stomatitis, PFAPA and Behçet disease, and Aydin et al. [8] compared these phenotypes clinically in a paediatric rheumatology cohort. Our study instead used recurrent oral aphthosis as the common referral phenotype and related baseline features to eventual diagnostic category.

The inverse fever/pharyngitis associations reflect the predominance of PFAPA in Group 1, whereas the genital aphthosis signal was predominantly Behçet-related: 22 of 23 Group 2 patients with genital aphthosis had Behçet disease or Behçet-like disease. After exclusion of Behçet spectrum diagnoses, only one exposed Group 2 patient remained, rendering the corresponding regression estimates highly imprecise and largely uninformative; these estimates should therefore not be interpreted as evidence that the association was absent. Although all analysed manifestations preceded final diagnostic assignment, incorporation bias remains because these clinical features also contribute to diagnostic reasoning.

Articular involvement was borderline in the full-cohort and Firth models and showed the most consistent positive association across diagnosis-exclusion sensitivity analyses, reaching statistical significance after exclusion of PFAPA and after simultaneous exclusion of PFAPA and Behçet spectrum diagnoses. This may represent a less diagnosis-specific systemic signal, although arthralgia and objective arthritis could not be separated retrospectively.

HLA-B51 positivity was more frequent in Group 2 among tested patients, consistent with its known association with Behçet disease [9]. Testing was driven by clinical suspicion and was therefore subject to indication bias. Importantly, HLA-B51 status was not used as a criterion for final diagnostic assignment, including Behçet-like disease, reducing concern that the observed association resulted from formal incorporation of HLA-B51 into the diagnostic definition. Nevertheless, because testing was selective, the magnitude of this association should be interpreted cautiously. CRP/ESR was also incompletely and non-uniformly sampled; lack of an independent association should not be interpreted as lack of clinical value.

A study strength is the temporal separation between baseline assessment and final diagnostic assignment. The relatively large referral cohort also permitted sparse data and diagnosis exclusion sensitivity analyses.

Limitations include incorporation bias, clinical heterogeneity of the two groups, dominance of PFAPA and Behçet spectrum diagnoses, and heterogeneous follow-up duration. Clinical reassessments were performed every 3–6 months, but exact individual follow-up duration could not be reliably reconstructed; later evolution of paediatric Behçet phenotypes may therefore have been missed. Patient-level demographic variables were not retained in the analytic dataset available for this revision; therefore, group-specific age-at-onset and sex distributions and age at the index rheumatology assessment could not be reliably reconstructed, precluding assessment of whether age differences contributed to the observed case mix. Other longitudinal variables, including diagnostic delay and family history, could not be reliably reconstructed. CRP/ESR and HLA-B51 were incompletely and selectively assessed.

Clinically, associated manifestations should be interpreted in context rather than converted into a diagnostic rule. Fever and pharyngitis are not reassuring in isolation, and genital aphthosis requires careful assessment despite its Behçet-driven association here. Longer prospective follow-up is needed to test generalisability.

## Conclusion

In children referred for recurrent oral aphthosis, baseline clinical features were associated with the eventual diagnostic category. The strongest PFAPA- and Behçet-related signals were substantially case-mix dependent, whereas articular involvement showed the most consistent positive association across sensitivity analyses. These findings are descriptive and require prospective validation before clinical use.

## Supplementary Information

Below is the link to the electronic supplementary material.

ESM 1 (DOCX 30.6 KB)

## Data Availability

No datasets were generated or analysed during the current study.

## Abbreviations

aOR: Adjusted odds ratio
CAPS: Cryopyrin-associated periodic syndromes
CI: Confidence interval
CVID: Common variable immunodeficiency
FMF: Familial Mediterranean fever
HLA-B51: Human leukocyte antigen B51
ICBD: International criteria for Behçet disease
MKD: Mevalonate kinase deficiency
OR: Odds ratio
PEDBD: Paediatric Behçet disease
PFAPA: Periodic fever, aphthous stomatitis, pharyngitis and cervical adenitis
SLE: Systemic lupus erythematosus
