# Supplementary material for: Oral aphthosis in children: baseline clinical features associated with diagnostic categories in a single-centre retrospective cohort
Source: Eur J Pediatr. 2026 Sep 15;185(10):749. doi: 10.1007/s00431-026-07405-4 (PMC13578049; doi:10.1007/s00431-026-07405-4)
Supplement: Supplementary file 1 [file 431_2026_7405_MOESM1_ESM.docx]

Supplementary Table S1. Exploratory HLA-B51 testing and positivity analyses

**Panel A. HLA-B51 status by diagnostic group**

| **HLA-B51 status** | **Total tested** | **Group 1** | **Group 2** | **OR (95% CI)** | **p value** |
| --- | --- | --- | --- | --- | --- |
| Positive | 36/218 (16.5%) | 7/147 (4.8%) | 29/71 (40.8%) | 13.81 (5.65-33.78) | <0.001 |
| Negative | 182/218 (83.5%) | 140/147 (95.2%) | 42/71 (59.2%) | Reference | -- |

**Panel B. HLA-B51 testing by final diagnosis**

| **Final diagnosis** | **Total n** | **HLA-B51 tested n (%)** | **HLA-B51 positive n/tested (%)** |
| --- | --- | --- | --- |
| PFAPA | 169 | 89 (52.7%) | 4/89 (4.5%) |
| Behçet-like disease | 50 | 23 (46.0%) | 13/23 (56.5%) |
| Recurrent fever | 33 | 7 (21.2%) | 0/7 (0.0%) |
| Recurrent oral aphthosis | 30 | 21 (70.0%) | 2/21 (9.5%) |
| Behçet disease | 30 | 24 (80.0%) | 12/24 (50.0%) |
| Recurrent infections | 26 | 22 (84.6%) | 0/22 (0.0%) |
| Familial Mediterranean fever | 21 | 9 (42.9%) | 2/9 (22.2%) |
| Juvenile idiopathic arthritis | 6 | 5 (83.3%) | 0/5 (0.0%) |
| Coeliac disease | 5 | 1 (20.0%) | 0/1 (0.0%) |
| Mevalonate kinase deficiency | 4 | 2 (50.0%) | 0/2 (0.0%) |
| Articular pain | 3 | 3 (100.0%) | 1/3 (33.3%) |
| Common variable immunodeficiency | 3 | 0 (0.0%) | -- |
| Suspected FMF | 3 | 0 (0.0%) | -- |
| IgA deficiency | 2 | 1 (50.0%) | 0/1 (0.0%) |
| Primary Raynaud phenomenon | 2 | 2 (100.0%) | 0/2 (0.0%) |
| Cutaneous lupus erythematosus | 2 | 0 (0.0%) | -- |
| PFAPA-like phenotype | 2 | 2 (100.0%) | 0/2 (0.0%) |
| Cutaneous vasculitis | 2 | 2 (100.0%) | 0/2 (0.0%) |
| Indeterminate colitis | 1 | 0 (0.0%) | -- |
| Connective tissue disease | 1 | 1 (100.0%) | 0/1 (0.0%) |
| Cryopyrinopathy | 1 | 0 (0.0%) | -- |
| Erythromelalgia | 1 | 1 (100.0%) | 0/1 (0.0%) |
| Secondary Raynaud phenomenon | 1 | 1 (100.0%) | 0/1 (0.0%) |
| Fibromyalgia | 1 | 0 (0.0%) | -- |
| Hypogammaglobulinaemia | 1 | 0 (0.0%) | -- |
| Crohn disease | 1 | 1 (100.0%) | 1/1 (100.0%) |
| IgA vasculitis/Henoch-Schönlein purpura | 1 | 0 (0.0%) | -- |
| TINU syndrome | 1 | 0 (0.0%) | -- |
| TRAPS | 1 | 1 (100.0%) | 1/1 (100.0%) |

**Panel C. Baseline characteristics of HLA-B51-tested versus untested patients**

| **Characteristic** | **Tested (n=218)** | **Untested (n=186)** | **p value** |
| --- | --- | --- | --- |
| Coexistence of minor and major aphthae | 10/218 (4.6%) | 7/186 (3.8%) | 0.681 |
| Recurrent fever | 151/218 (69.3%) | 153/186 (82.3%) | 0.003 |
| Genital aphthosis | 17/218 (7.8%) | 9/186 (4.8%) | 0.227 |
| Lymphadenopathy | 55/218 (25.2%) | 64/186 (34.4%) | 0.044 |
| Tonsillitis/pharyngitis | 125/218 (57.3%) | 117/186 (62.9%) | 0.255 |
| Arthralgia/arthritis | 72/218 (33.0%) | 67/186 (36.0%) | 0.528 |
| Abdominal pain | 78/218 (35.8%) | 79/186 (42.5%) | 0.169 |
| Bowel habit changes | 37/218 (17.0%) | 25/186 (13.4%) | 0.326 |
| Group 2 classification | 71/218 (32.6%) | 66/186 (35.5%) | 0.537 |

HLA-B51 testing was selective and based on clinical suspicion of Behçet disease rather than a uniform protocol. HLA-B51 status was not used as a criterion for final diagnostic assignment, including the working diagnosis of Behçet-like disease. Exploratory comparisons are descriptive. OR, odds ratio; CI, confidence interval.
